# Bayesian hyperparameter optimization improves scGPT fine-tuning for single-cell multi-omics integration

**DOI:** 10.1093/bioinformatics/btag374

**Published:** 2026-06-12

**Authors:** Darren Yu Jun Tay, Nguyen Quoc Khanh Le, Matthew Chin Heng Chua

**Affiliations:** Science Research Programme, Catholic Junior College, Singapore 297822, Singapore; AIBioMed Lab, Taipei Medical University, Taipei 110, Taiwan; AIBioMed Lab, Taipei Medical University, Taipei 110, Taiwan; In-Service Master Program in Artificial Intelligence in Medicine, College of Medicine, Taipei Medical University, Taipei 110, Taiwan; Translational Imaging Research Center, Taipei Medical University Hospital, Taipei 110, Taiwan; Department of Biomedical Informatics, Yong Loo Lin School of Medicine, National University of Singapore, Singapore 119615, Singapore

## Abstract

**Motivation:**

Foundation models such as scGPT have demonstrated strong potential for single-cell multi-omics integration; however, their downstream performance is highly sensitive to hyperparameter selection. Manual fine-tuning remains computationally expensive, dataset-dependent, and often irreproducible. Despite the increasing adoption of foundation models in single-cell analysis, systematic strategies for robust hyperparameter optimization remain underexplored.

**Results:**

We developed a Bayesian optimization framework based on Tree-structured Parzen Estimators (TPE) for automated fine-tuning of scGPT and evaluated its performance on two benchmark bone marrow mononuclear cell (BMMC) multi-omics datasets, including CITE-seq and GSE194122 datasets. Across datasets, Bayesian optimization consistently improved biological conservation and batch integration metrics compared with default scGPT configurations. On the original BMMC benchmark, optimization improved AvgBIO from 0.59 to 0.67 and PCR from 0.33 to 0.52. On the GSE194122 dataset, the default configuration exhibited unstable convergence and weak biological preservation (AvgBIO = 0.19; ARI = 0.007), whereas Bayesian optimization substantially improved integration performance (AvgBIO = 0.60; ARI = 0.63) while reducing validation loss from 137 to 47.1. These findings demonstrate substantial dataset-specific sensitivity of scGPT fine-tuning and highlight the importance of automated optimization for stable deployment across heterogeneous multi-omics datasets. Our study demonstrates that Bayesian optimization provides an effective and reproducible strategy for stabilizing scGPT fine-tuning across diverse single-cell multi-omics datasets. Rather than introducing a new integration architecture, this work emphasizes the importance of systematic optimization for improving robustness and reproducibility of foundation-model applications in computational biology.

**Availability and implementation:**

Our model and dataset are freely available at: https://github.com/daren642/scGPT_multiomic_tuning.

## 1 Introduction

Large language models (LLMs) have emerged as powerful tools across research domains, with scGPT representing an important advance in single-cell transcriptomics and multi-omic integration (Cui *et al*. 2024). Designed to address the fragmented nature of single-cell studies (Angerer *et al*. 2017), where models are often tailored to specific research goals (Ji *et al*. 2021, Liu *et al*. 2021, Oller-Moreno *et al*. 2021), scGPT uses unsupervised generative pre-training on a large-scale dataset comprising 33 million scRNA-seq records from cell atlases (Regev *et al*. 2017). Unlike general-purpose LLMs developed for natural language processing, scGPT focuses on learning gene-level embeddings and biological relationships, enabling its application across a broad range of transcriptomic and integrative analyses. These applications include transcriptomics (Yan *et al*. 2024), cell type annotation (Fang *et al*. 2024), multi-omic integration (Rodov *et al*. 2025), and related tasks. While this study focuses specifically on multi-omic integration, prior work has demonstrated the practical utility of scGPT in diverse biological and clinical research settings (Tan *et al*. 2024, Chang *et al*. 2025).

The core functionality of scGPT lies in its ability to process multi-omic inputs through a transformer architecture, converting high-dimensional molecular features into embeddings that capture meaningful biological relationships. The model leverages a self-attention mechanism to maintain context across input tokens (Vaswani *et al*. 2017). These embeddings are first learned through large-scale pre-training, such as datasets curated in the CELLxGENE collection (Program *et al*. 2025), and can subsequently be fine-tuned with labeled data for specific downstream applications.

A critical component of adapting foundation models for downstream tasks is hyperparameter tuning. While parameters such as weights and biases are optimized during backpropagation, hyperparameters—such as learning rate, batch size, or model depth—must be predefined and can substantially influence performance (Owen 2022). In practice, these settings are often selected manually or through heuristic trial-and-error, which is time-consuming and may lead to suboptimal or irreproducible results, particularly for computationally intensive models such as scGPT.

Traditional hyperparameter optimization approaches include grid search, which systematically evaluates all combinations within a predefined space, and randomized search (Bergstra and Bengio 2012), which samples configurations more efficiently. However, both strategies suffer from the curse of dimensionality (COD) (Keogh and Mueen 2011), where computational cost grows rapidly as the number of tunable parameters increases. To address this limitation, Bayesian optimization has emerged as an effective alternative, especially for deep learning workflows (Hanifi *et al*. 2024); (Kumar Lilhore *et al*. 2024). By adaptively balancing exploration and exploitation, Bayesian optimization can identify promising configurations using fewer evaluations. In this study, we use Tree-structured Parzen Estimators (TPE) (Ozaki *et al*. 2022), a Bayesian optimization method, to enable efficient and automated hyperparameter tuning of scGPT. Our goal is to systematically improve fine-tuning performance while reducing manual effort for multi-omic integration tasks.

Several recent studies have explored automated or deep learning-based approaches for single-cell integration and analysis (Qi *et al*. 2020, 2021). In contrast, our work focuses specifically on systematic hyperparameter optimization for an existing foundation model rather than proposing a new integration architecture.

## 2 Materials and methods

### 2.1 Overview

We developed a hyperparameter optimization framework for scGPT, a large transformer foundation model for single-cell data, by embedding TPE Bayesian optimization loop into the model fine-tuning pipeline (Kushner 1964). Our objective was to identify hyperparameter settings that minimize validation loss and improve downstream biological and batch-integration metrics (ARI, NMI, PCR, AvgBIO) under realistic computational constraints typical of academic research environments. We retained the pretrained scGPT architecture where required for checkpoint compatibility, fixing the hidden dimension (512) and number of attention heads (8). We also kept key training settings constant across trials (dropout=0.20, StepLR γ=0.95, and mixed-precision training enabled) to isolate the effects of the tuned hyperparameters. FlashAttention/fast-transformer kernels were not used in our environment.

### 2.2 Bayesian optimization background

Bayesian optimization (BO)—also called sequential model-based optimization—addresses expensive black-box functions by building a probabilistic surrogate of the objective and using an acquisition function to choose the next evaluation point (Kushner 1964). Classic BO approaches trace to early work on stochastic search [e.g. Kushner (1964)] and subsequent formalizations by Mockus and others in the context of global optimization of costly functions (Močkus 1974, Elder 1992, Stuckman 2002). In our setting the black-box function f(x) is the validation loss (or another performance proxy) obtained when scGPT is fine-tuned under hyperparameter configuration *x* drawn from a search space X.

We formalize the optimization problem as:


(1) $$x^{*}=\arg\underset{x\in X}{\min}f(x),$$

where lower f(x) indicates better validation performance (Shahriari *et al*. 2015, Wang *et al*. 2023). Traditional exhaustive (grid) or uninformed (random) search becomes prohibitively expensive as the dimensionality and training cost increase—conditions common to large transformer models. BO reduces the number of expensive function evaluations required to identify high-performing configurations.

#### 2.2.1 Surrogate models

Many BO implementations use Gaussian processes (GPs) to model f(x). A GP prior,


(2) $$f(x)∼GP(m(x),k(x,x^{\prime })),$$

is specified by mean function m(x) (often 0) and covariance kernel k(.,.) (e.g. squared exponential or RBF). With noisy observations $y=f(x)+\varepsilon ,\;\varepsilon ∼N(0,\sigma_{\varepsilon }^{2})$, the joint distribution of observed and unobserved function values remains multivariate normal, yielding closed-form posterior predictive mean $\mu_{*}$ and covariance $\Sigma_{*}$:


(3) $$\mu_{*}=K_{*,X}{\left(K_{X,X}+\sigma_{\varepsilon }^{2}I\right)}^{-1}y,$$

(4) $$\Sigma_{*}=K_{*,*}-K_{*,X}{\left(K_{X,X}+\sigma_{\varepsilon }^{2}I\right)}^{-1}K_{X,*}.$$

GPs provide calibrated uncertainty but can degrade computationally and statistically in high-dimensional, heterogeneous, mixed-type hyperparameter spaces—a common scenario when tuning large neural models.

#### 2.2.2 Acquisition functions

Given a surrogate predictive distribution, an acquisition function a(x) trades off exploration (high uncertainty) and exploitation (low predicted loss). The next configuration is:


(5) $$x_{\text{next}}=\arg\underset{x\in X}{\max}a(x)$$

Common choices include Expected Improvement (EI), Probability of Improvement (PI), and Upper/Lower Confidence Bounds. We use an EI-style criterion within the TPE formulation (Bousquet *et al*. 2011, Shahriari *et al*. 2015).

### 2.3 Tree-structured Parzen Estimators (TPE) for hyperparameter search

Because our search space mixes continuous, discrete, and categorical parameters and can include hierarchical dependencies, we adopt the TPE algorithm [as implemented in Hyperopt (Bergstra *et al*. 2015)] rather than a standard GP surrogate (Bergstra *et al*. 2011). TPE is a density estimation approach that models the inverse conditional p(x∣y) instead of p(y∣x) (Claret *et al*. 2024). Observed trials are split at a performance quantile threshold $y^{*}$ (often the γ-quantile of losses):

-
$\ell (x)=p\left(x|y<y^{*}\right)$ describes configurations associated with good (low) losses.
-
$g(x)=p\left(x|y\geq y^{*}\right)$ describes configurations associated with worse losses.

Both densities are estimated nonparametrically (e.g. kernel density estimators) in a tree-structured manner that naturally handles conditional / hierarchical hyperparameters (Akber *et al*. 2024). Candidate points are proposed by sampling from ℓ(x) and scored by the ratio ℓ(x)/g(x); values that are common under ℓ but rare under *g* receive high Expected Improvement. In practice, Hyperopt samples many candidates from ℓ(x), evaluates the *EI*-proportional criterion, and selects the best for expensive evaluation (Zhou *et al*. 2021, Chen *et al*. 2022).

Formally, letting $\gamma =P(y<y^{*})$, marginalizing over *y* yields


(6) p(x)=γ ℓ(x)+(1−γ)g(x),

and the *EI* criterion is proportional to


(7) $$\text{EI}(x)\propto {\left(\gamma +\frac{1-\gamma }{\ell (x)/g(x)}\right)}^{-1},$$

so maximizing EI corresponds to selecting *x* with large g(x)/ℓ(x) (good-region density relative to bad-region density).

#### Hyperparameter search space

We tuned four key hyperparameters: the number of transformer layers (nlayer  ∈ 2,3,4,5,6,7), learning rate (uniform prior over [1e-5, 1e-3]), training epochs (∈ 20,30,40), and batch size (∈ 16,32). Defaults followed the scGPT baseline (e.g. nlayer=4, learning rate=1e-3, batch size=16), and the best configuration selected by TPE was nlayer=7, learning rate=3.21×10^−4^, epochs=40, and batch size=32.

### 2.4 scGPT fine-tuning and TPE search protocol

We fine-tuned the publicly released scGPT_human checkpoint under varying hyperparameter configurations proposed by TPE. Each trial:

- Configuration sampling: Draw x = {nlayers, learning_rate, epochs, batch_size} from the TPE search space (Table 1).
- Model instantiation: Expand pretrained token embeddings to match dataset vocabulary; initialize new layers when depth increases.
- Training: Fine-tune for the sampled number of epochs using Adam (eps scaled to mixed-precision setting), StepLR schedule (γ = 0.95/epoch), and loss terms enabled per configuration (GEP, GEPC, DAR).
- Validation: Track validation loss each epoch; retain the checkpoint with lowest validation loss for downstream metrics.
- Logging: Record metrics (ARI, NMI, PCR, AvgBIO), wall-clock time, and resource use to Weights & Biases.

**Table 1:** Complete specification of the hyperparameter search used to optimize scGPT fine-tuning with Tree-structured Parzen Estimators (TPE).

Name	Type	Range/choices	Prior (Hyperopt)	Default	Best (TPE)
nlayers (Transformer encoder layers)	D	2,3,4,5,6,7	hp.choice ("n_layers", [2,3,4,5,6,7])	4	7
learning_rate	C	[1e−5, 1e−3]	hp.uniform ("learning_rate", 1e−5, 1e−3)	1e−3	3.21e−4
epochs	D	20,30,40	hp.choice ("epochs", [20,30,40])	25	40
batch_size	D	16,32 (extendable)	hp.choice ("batch_size", [16,32])	16	32

#### 2.4.1 Hyperparameter tuning

Key hyperparameters tuned included the number of Transformer encoder layers, batch size, training epochs, learning rate, and schedule ratio. To ensure compatibility with the released scGPT_human checkpoint, the hidden dimension and number of attention heads were fixed. TPE algorithm from the Hyperopt library was used to automate the search. Cross-Entropy Loss served as the evaluation metric (Boudiaf *et al*. 2020, Mao *et al*. 2023):


(8) $$H(p,q)=-\sum_{i}p(i)\;\text{log}\;q(i)$$

where p(i) is the true distribution and q(i) the estimated one (Stanton *et al*. 2022, Lilhore *et al*. 2024). Validation loss was computed after each trial, and the best-performing configuration was selected.

We defined the initial search space to balance coverage and feasibility. Ranges for learning rate, batch size, number of layers, and epochs were centered around scGPT’s recommended/default settings and values commonly used in transformer fine-tuning for single-cell foundation models. We excluded regimes known to be unstable or infeasible under our compute constraints (e.g. excessively large learning rates or batch sizes that exceed GPU memory). This focused design reduces the number of costly evaluations required for Bayesian optimization. Expanding the space to include additional parameters (e.g. dropout, weight decay, alternative schedulers, or architecture widths) would substantially increase the trial budget needed for reliable optimization and therefore the total runtime.

The search included 10 randomly initialized trials followed by 20 TPE-guided trials (30 in total). All trials used a fixed random seed (42) for reproducibility, and the full TPE run was repeated across five seeds to assess robustness. Table 1 provides complete definitions, defaults, and best TPE-identified values. Convergence analysis revealed diminishing returns after approximately 25 trials. During optimization, we enabled reconstruction and alignment objectives (GEP/GEPC and DAR) while disabling supervised cell-type classification (CLS) to avoid label leakage during tuning.

#### 2.4.2 Fixed training/objective settings

Unless otherwise stated: hidden size = 512; nhead = 8; dropout = 0.2; mixed precision enabled; GEP, GEPC, and DAR objectives active; CLS and ESC disabled; domain-specific batchnorm disabled; input tokens binned to 51 levels; HVG selection = 1200 RNA features; modality tokens enabled for RNA versus protein.

### 2.5 Dataset and preprocessing

We utilized the bone marrow mononuclear cells (BMMC) dataset obtained via Cellular Indexing of Transcriptomes and Epitopes (CITE-seq), which includes paired RNA and surface protein abundance data. This dataset contains multiple batches grouped by donor origin. Each cell provides simultaneous RNA and protein measurements, making it ideal for evaluating multi-omic integration. The pre-trained whole-body model of scGPT was fine-tuned on this dataset. Hyperparameter tuning code is provided in the accompanying file "Hyperparameter_tuning_best".

To further demonstrate the effectiveness of Bayesian optimization, a large-scale human bone marrow single-cell multi-omics dataset GSE194122 (Luecken *et al*. 2021) designed primarily to benchmark methods for integrating RNA, chromatin accessibility, and protein data across batches and modalities was used. The dataset comprised of single-cell multiomics data collected from bone marrow mononuclear cells of 12 healthy human donors.

### 2.6 Biological marker audit and cluster diagnostics

To interpret clustering differences biologically, we curated reference marker panels (RNA ± ADT) mapping canonical lineage markers to high-level cell-type labels. Panels were dataset-specific: e.g. CD3E/TRAC (T), MS4A1/CD79A (B), LST1/LYZ (monocyte), NKG7/GNLY (NK) for immune datasets; appropriate epithelial / endothelial / mesenchymal markers for tissue datasets. RNA counts were log-normalized; ADT values CLR-transformed consistent with preprocessing.

For each method’s clustering result, we computed marker scores = mean *z*-score across genes in a lineage panel for every cluster. The lineage with the highest marker score became the marker label for that cluster. We quantified:

- Purity: fraction of cells in a cluster whose ground-truth annotation matches its marker label.
- Lineage recovery: fraction of cells of a given lineage captured in the cluster with the highest marker score for that lineage.
- Over/Under-Splitting Index (OUI): number of clusters containing ≥5% of a lineage’s cells (threshold tunable) relative to the ideal of 1; OUI>1 indicates over-splitting; OUI<1 indicates merging.
- Confusion matrix: counts (or row-normalized frequencies) of ground-truth lineage × cluster marker label, used to visualize misassignments.
- Differential expression: For discordant clusters across methods we performed Wilcoxon rank-sum tests (RNA) and two-sample t-tests (ADT); significant genes/antibodies (FDR<0.05; |logFC|>0.25) are reported.
- Batch-mixing versus purity tradeoff: For each cluster we computed a cluster-restricted PCR (PCR_cluster) and plotted PCR_cluster versus purity to visualize integration/biology tradeoffs.

### 2.7 Statistical analysis

We evaluated stochastic variability by repeating each method (default scGPT, TPE-tuned scGPT, and baseline comparators) [*n*= 3] independent runs with different random seeds using identical train/validation splits. For each seed we recorded ARI, NMI, PCR, and AvgBIO. Paired differences (tuned—comparator) were tested with the Wilcoxon signed-rank test (primary, robust for small *n*); because *n* is modest, *P*-values are interpreted cautiously and supplemented with rank-biserial effect size and bias-corrected bootstrap 95% confidence intervals (10 000 resamples of the seed-level differences). False discovery rate (Benjamini–Hochberg) was controlled within each metric family across dataset/method comparisons. These statistical results replace informal descriptive comparisons in the initial submission.

### 2.8 Evaluation metrics

The overall performance was further assessed using AvgBIO, an aggregate metric that evaluates biological conservation. AvgBIO is calculated as:


(9) $$\text{AvgBIO}=\frac{{\text{ARI}}_{\text{cell}}+{\text{NMI}}_{\text{cell}}+{\text{ASW}}_{\text{cell}}}{3}$$

where:

- Adjusted Rand Index (ARI): Measures the agreement between annotated labels and Louvain clusters. ARI ranges from 0 to 1, with higher scores indicating better alignment.
- Normalized Mutual Information (NMI): Quantifies the overlap between cell type labels, with values ranging from 0 to 1. Higher scores indicate better matches. Louvain clustering was performed at resolutions ranging from 0.1 to 2, in increments of 0.1.
- Average Silhouette Width (ASW): Assesses cluster separability, ranging from −1 to 1. A score of 1 indicates well-separated clusters.

### 2.9 Hardware, software

Experiments were run on a system with an 11th Gen Intel Core i7-11700KF CPU @ 3.60 GHz, NVIDIA RTX 3060 Ti GPU, 16 GB RAM, and Windows 10 OS. Despite being consumer-grade hardware, this setup enabled comprehensive tuning, though it lacks the efficiency of high-end GPUs like the NVIDIA A100.

Dependency conflicts required specific library versions: anndata 0.10.8, torch 2.2.2+cu118, torchtext 0.18.0+cpu, torchvision 0.17.2, numpy 1.24.1, and scipy 1.13.1. Notably, the FlashAttention module required for efficient attention computation (>v1.0.5) was incompatible with scGPT’s version constraints (<v1.0.5), limiting potential performance. Nevertheless, the study demonstrated the viability of scGPT fine-tuning using TPE even under such constraints. Batch size was constrained by GPU memory; in our setting, batch size 32 was feasible with hidden size 512 and 8 attention heads, whereas larger batches would exceed the available memory budget.

## 3 Results and discussion

### 3.1 Validation performance

**Figure 1:**
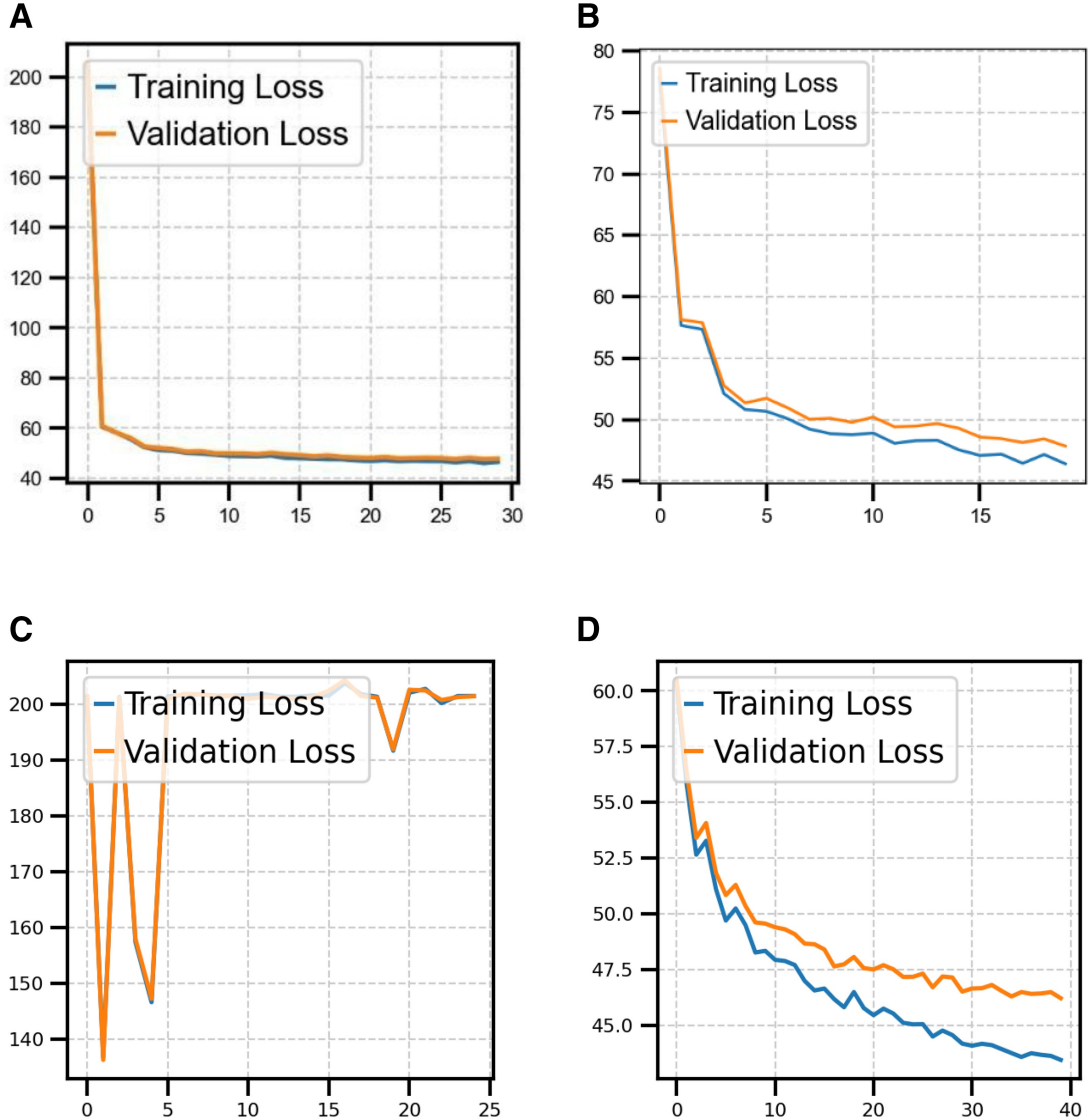
Training dynamics of scGPT fine-tuning under default and Bayesian-optimized configurations across two benchmark datasets. (A) Original BMMC dataset using default hyperparameters. (B) Original BMMC dataset after Bayesian optimization. (C) GSE194122 dataset using default hyperparameters. (D) GSE194122 dataset after Bayesian optimization. Training and validation cross-entropy losses are plotted across epochs. Bayesian optimization produced faster and more stable convergence with substantially lower validation loss across both datasets. In contrast, the default configuration on GSE194122 exhibited unstable optimization behavior characterized by oscillatory losses and persistently poor convergence.

Fine-tuning reduced the validation loss by approximately two points compared with the default scGPT configuration (Fig. 1), indicating improved optimization under the tuned settings. AvgBIO, a composite biological conservation score, increased from 0.59 (default) to 0.67 after tuning (+8%). The fine-tuned model also achieved higher biological conservation than Seurat v4 (AvgBIO = 0.60).

Across random seeds, training and validation losses exhibited similar convergence trends without evident divergence, suggesting stable training on the evaluated benchmark. Improvements were consistent across runs and supported by statistical testing with large effect sizes (|r_rb_| ≥ 0.6). To maintain computational feasibility, the search space was restricted to the most influential hyperparameters, as described in the Methods.

### 3.2 Performance across seeds and datasets

Across five random seeds on the BMMC dataset, the fine-tuned scGPT consistently improved clustering and integration metrics, including ARI (+0.07), NMI (+0.05), and AvgBIO (+0.04). Batch correction, measured by PCR (Principal Component Regression), improved by +0.19. Paired Wilcoxon signed-rank tests confirmed significant gains for ARI and AvgBIO (FDR-adjusted *q* < 0.05), with large effect sizes ($|r_{\text{rb}}|\geq 0.6$), indicating that improvements were consistent rather than driven by isolated runs (Table 2).

**Table 2:** Comparison of default and tuned-hyperparamter results.^a^

	BMMC			GSE194122		
	Default	Fine-tuned	Difference	Default	Fine-tuned	Difference
Validation loss	49.8	47.8	−2	137	47.1	−89.9
Avg_bio	0.59	0.67	+0.08	0.19	0.60	+0.41
ARI_cluster	0.48	0.66	+0.18	0.007	0.63	+0.62
NMI_cluster	0.64	0.70	+0.06	0.13	0.68	+0.55
PCR_batch	0.33	0.52	+0.19	0	0.54	+0.53
epoch	25	20	−5	25	40	+15

a Avg_bio: biological relevance; ARI_cluster: clustering accuracy; NMI_cluster: clustering-label alignment; PCR_batch: batch effect correction.

**Figure 2:**
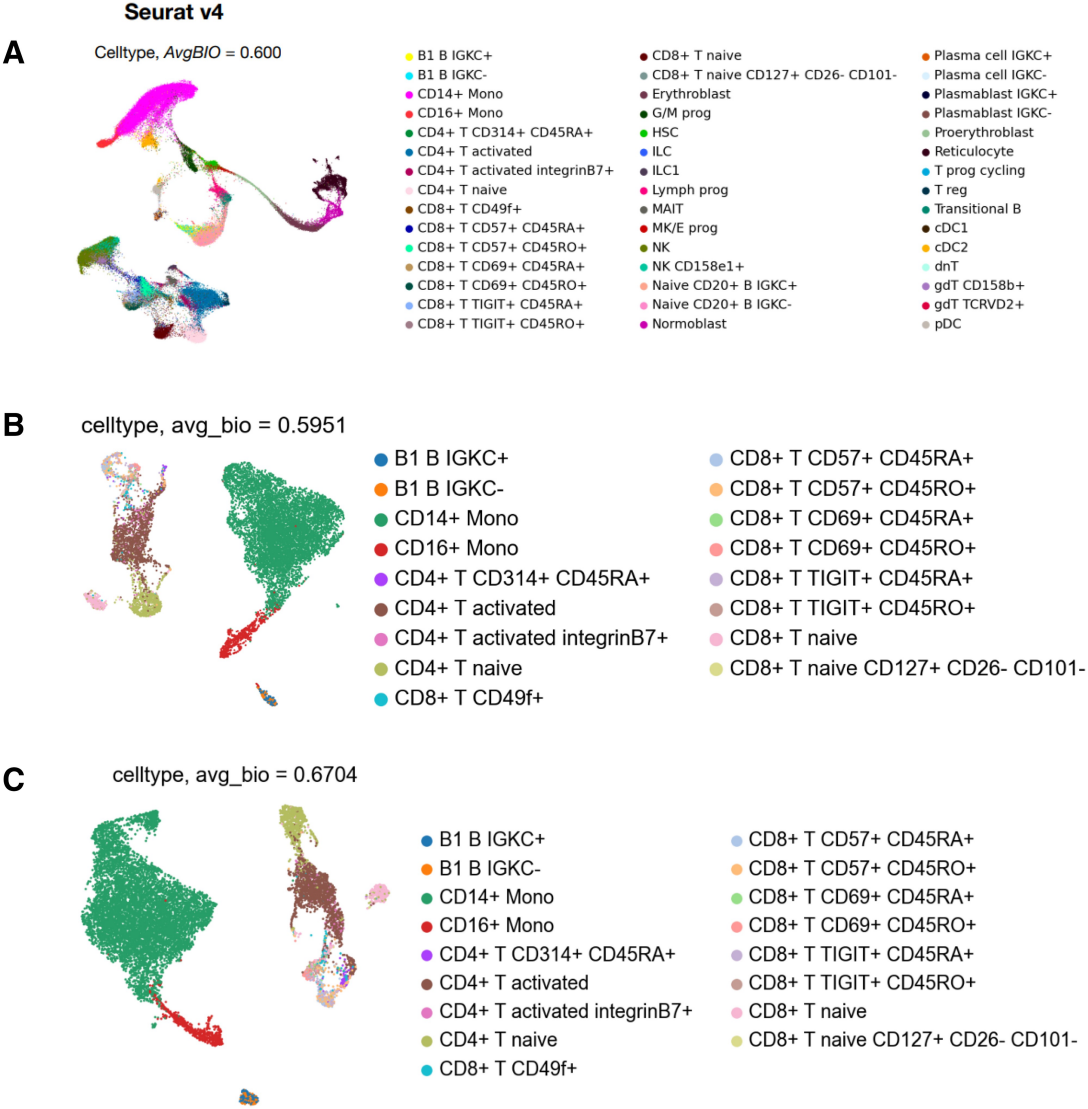
UMAP visualization of integrated embeddings on the BMMC CITE-seq dataset. Cells are colored by annotated cell types (top row) and sequencing batches (bottom row). Compared with the default configuration, the optimized model exhibits clearer cell-type separation and improved batch mixing. UMAPs are presented for qualitative illustration only; statistical conclusions are based on quantitative integration metrics (AvgBIO, PCR, ARI, and NMI). (A) Seurat v4 baseline; (B) scGPT prior to hyperparameter tuning; (C) scGPT after fine-tuning with Bayesian optimization (TPE).

UMAP visualizations for BMMC CITE-seq (Fig. 2A–C) dataset provide qualitative illustrations of improved cell-type separation and batch mixing under the tuned configuration. As UMAP is primarily descriptive, conclusions are based on the quantitative integration metrics reported above.

### 3.3 Dataset-specific sensitivity of scGPT fine-tuning

To evaluate whether the optimized hyperparameters generalized beyond the original benchmark dataset, we performed additional experiments on the GSE194122 multi-omics dataset. Unlike the original BMMC benchmark, the default scGPT configuration exhibited unstable optimization behavior characterized by oscillatory validation loss and weak biological conservation.

**Figure 3:**
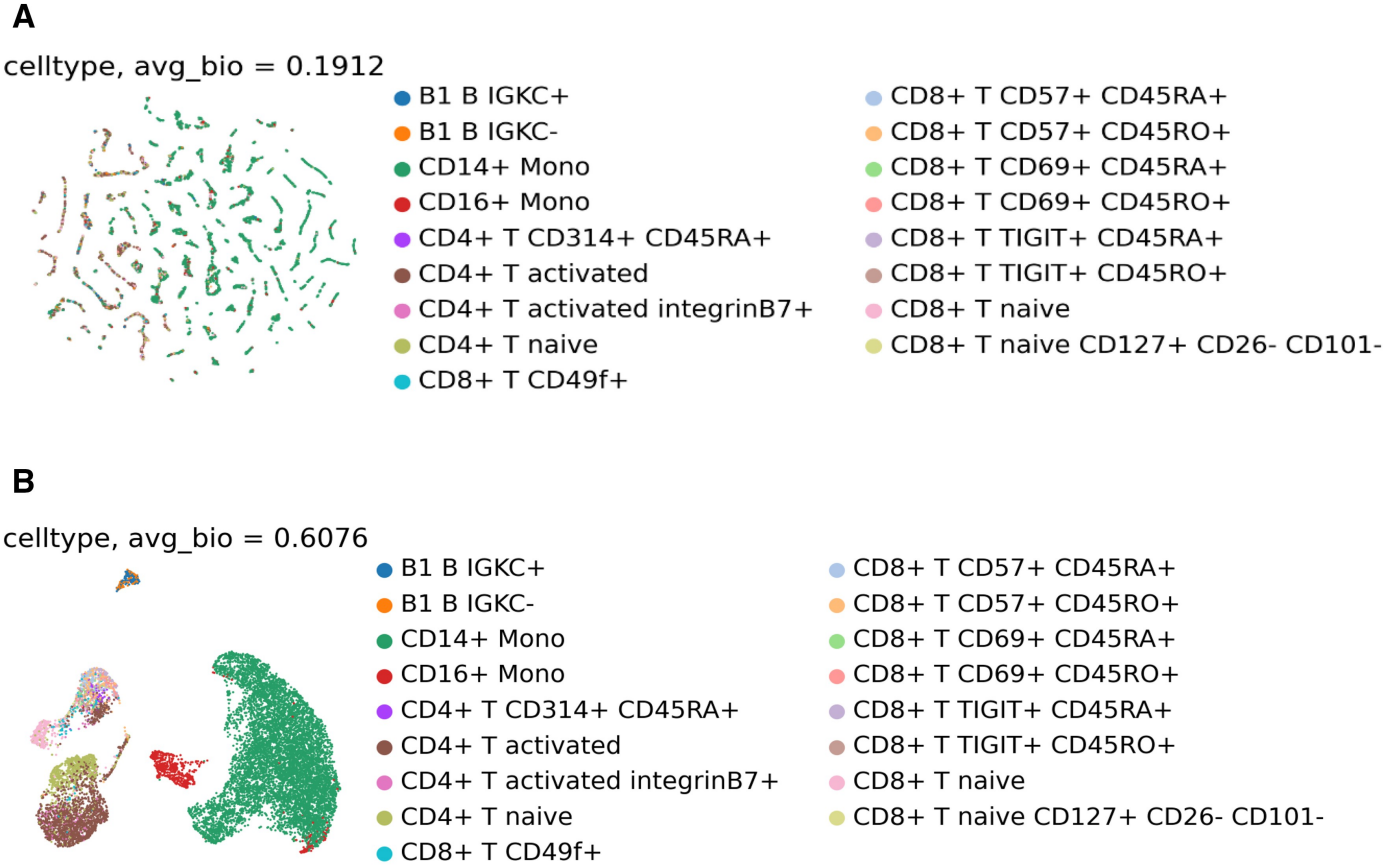
UMAP visualization of integrated embeddings on the GSE194122 multi-omics benchmark dataset before and after Bayesian hyperparameter optimization of scGPT. Cells are colored according to annotated cell types. (A) Default scGPT configuration prior to Bayesian optimization. The default setting exhibits poor biological structure preservation, fragmented clustering patterns, and weak separation between cell populations, consistent with the low AvgBIO score (0.1912). (B) Bayesian-optimized scGPT configuration using Tree-structured Parzen Estimators (TPE). Following optimization, cell populations become substantially more compact and biologically coherent, with improved cluster separation and increased biological conservation (AvgBIO = 0.6076).

Under default settings, the model achieved poor integration performance (AvgBIO = 0.19, ARI = 0.007, PCR = 0), indicating ineffective preservation of biological structure and inadequate batch correction. In contrast, Bayesian optimization substantially improved convergence stability and downstream integration quality, reducing validation loss from 137 to 47.1 while improving AvgBIO to 0.60 and ARI to 0.63 (Fig. 3 and Table 2).

One likely contributing factor was the high default learning rate (0.003), which may produce excessively large parameter updates during transformer fine-tuning. Bayesian optimization instead identified a substantially lower learning rate (0.0003), enabling smoother convergence dynamics and improved preservation of biological structure. These findings suggest that scGPT fine-tuning may be highly sensitive to dataset-specific hyperparameter configurations, highlighting the importance of automated optimization strategies for robust deployment across heterogeneous multi-omics datasets.

### 3.4 Additional performance metrics

Beyond AvgBIO, the tuned configuration improved several complementary integration metrics. The ARI increased by 0.18, indicating better agreement between predicted clusters and reference annotations. NMI improved by 0.06, and PCR, which quantifies batch-effect removal, increased by 0.19. Together, these results further demonstrate improved clustering quality and batch correction after Bayesian optimization (Liu *et al*. 2021, Rodov *et al*. 2025).

Notably, the magnitude of improvement observed on GSE194122 was substantially larger than that observed on the original BMMC benchmark. This suggests that default scGPT hyperparameters may not generalize consistently across heterogeneous datasets. The large improvements in ARI (+0.62) and AvgBIO (+0.41) further indicate that appropriate optimization is critical for preserving biological structure during foundation-model fine-tuning.

To assess the practical cost of optimization, we also report computational efficiency metrics, including (i) average time per epoch, (ii) average time per fine-tuning trial, and (iii) total wall-clock time for the full optimization budget. The difference between fine-tuned and default models is −9 minutes in total time and 15 s in time taken per epoch average.

### 3.5 Biological interpretation of clustering

To assess biological relevance, we conducted a marker-guided audit across Seurat v4, scGPT-default (Cui *et al*. 2024), and scGPT-TPE. Canonical RNA and ADT markers were used (e.g. CD3E/TRAC for T cells, MS4A1/CD79A for B cells, GNLY/NKG7 for NK cells). Clusters were labeled by majority-marker expression, and evaluated for purity and lineage recovery.

Cluster granularity was quantified using the Over/Under-Splitting Index (OUI). For example, CD4 T-cell OUI improved from 2.8 (Seurat) to 1.4 (scGPT-TPE), and B-cell OUI from 3.1 to 1.6. Confusion matrix analysis revealed Seurat split classical monocytes across donor batches, while scGPT-TPE preserved biological coherence and reduced batch effect.

Differential expression analysis showed Seurat’s B-cell splits were driven by Ig isotype levels without robust transcriptional separation. scGPT-TPE consolidated these into one biologically coherent B-cell cluster with plasmablast subclusters.

Moreover, a tradeoff analysis of batch mixing versus purity revealed scGPT-TPE clusters occupied a Pareto-optimal region—higher purity, lower PCR—across major lineages. Platelets remained challenging for all methods.

### 3.6 Limitations and future work

Several limitations should be acknowledged. First, evaluation was conducted on a single benchmark dataset (BMMC), and additional biological contexts such as tumors, inflamed tissues, and developmental trajectories were not examined. Consequently, cross-dataset generalization remains to be systematically validated in future studies.

Second, scGPT fine-tuning is computationally intensive. Hardware constraints limited the exploration of larger batch sizes, deeper architectures, and broader hyperparameter search spaces. Expanding these settings or adopting flash-attention-enabled and high-performance computing environments may further improve efficiency.

Third, the composite integration metric AvgBIO does not capture all aspects of biological structure, such as trajectory continuity or rare-cell detection. Incorporating complementary metrics (e.g. kBET, LISI, and trajectory conservation scores) would provide a more comprehensive evaluation. In addition, reliance on reference annotations introduces potential label noise, which may affect absolute performance estimates.

Finally, Bayesian optimization may favor dataset-specific configurations. Although consistent improvements were observed on the evaluated benchmark, transferability across diverse datasets requires further investigation and represents an important direction for future work.

## 4 Conclusion

We demonstrate that Bayesian hyperparameter optimization substantially improves the robustness and stability of scGPT fine-tuning for single-cell multi-omics integration. Across multiple benchmark datasets, automated optimization consistently improved convergence behavior, biological conservation, and batch integration quality while reducing dependence on manual tuning. Our findings further reveal substantial dataset-specific sensitivity in foundation-model fine-tuning, underscoring the importance of systematic optimization for reproducible deployment of large-scale foundation models in computational biology.

## Data Availability

Our model and dataset are freely available at: https://github.com/daren642/scGPT_multiomic_tuning.
